# COVID-19: Technology-Supported Remote Assessment of Pediatric Asthma at Home

**DOI:** 10.3389/fped.2020.00529

**Published:** 2020-09-08

**Authors:** Mattienne R. van der Kamp, Monique Tabak, Sophia E. J. A. de Rooij, Pieter P. E. van Lierop, Boony J. Thio

**Affiliations:** ^1^Department of Pediatrics, Medisch Spectrum Twente, Enschede, Netherlands; ^2^Department of Biomedical Signals and Systems, University of Twente, Enschede, Netherlands; ^3^Roessingh Research and Development, Enschede, Netherlands; ^4^Board of Directors, Medisch Spectrum Twente, Enschede, Netherlands; ^5^Department of Internal Medicine and Geriatrics, University of Groningen, University Medical Center Groningen, Groningen, Netherlands; ^6^Medical Director Pediatric Department, Medisch Spectrum Twente, Enschede, Netherlands

**Keywords:** COVID-19, child, telemedicine, asthma, technology, wearable electronic devices, self-management, delivery of healthcare

## Abstract

The COVID-19 crisis has pressured hospital-based care for children with high-risk asthma as they have become deprived of regular clinical evaluations. However, COVID-19 also provided important lessons about implementing novel directions for care. Personalized eHealth technology, tailored to the individual and the healthcare system, could substitute elements of hospital care and facilitate early and appropriate medical anticipation in response to imminent loss of control. This perspective article discusses new approaches to the clinical, organizational, and scientific aspects of the use of eHealth technology in pediatric asthma care in times of COVID-19, as illustrated by a case report of an acute asthma exacerbation possibly caused by COVID-19 infection.

## Introduction

### COVID-19 in Pediatric Asthma

Children of all ages can contract COVID-19 but appear to be less affected than adults (1, 2). In a systematic literature review, children accounted for 1–5% of diagnosed COVID-19 cases (3). Although severe cases have been reported, symptoms appear to be milder in children (2, 4).

The early transmission of COVID-19 was started by adults, which could be misunderstood as an indication of children being less infectious. However, the recent study of Jones et al. (5) shows that the viral loads in children do not differ significantly from those of adults, indicating that children may be as infectious as adults.

Because of the fewer reported cases of children with COVID-19 symptoms, it sometimes seems to be forgotten that a large number of children—those with serious underlying conditions, including chronic pulmonary diseases as moderate to severe asthma—are at a greater risk for a severe disease course (4). This accounts for many children as asthma is the most common chronic disease in childhood (6).

### The COVID-19 Crisis and Childhood Asthma Care

Acute asthma draws heavily on pediatric healthcare resources as it is one of the main causes of hospital visits. However, the COVID-19 outbreak has influenced the care of asthma children at risk in several ways, and a current challenge of great interest is how to continue high-quality care under these circumstances. As a result of the COVID-19 outbreak, scheduled clinical evaluations have been suspended and substituted by phone calls. From these telephonic consultations, we observe that the parents of these vulnerable children are afraid that their child contracts the COVID-19 virus either in or outside the hospital or that they transmit the virus themselves to their child.

Scheduled diagnostics in hospital facilities, such as lung function for monitoring the fluctuation of asthma, have also been restricted recently due to the risk of viral transmission (7). Pediatricians have extended medication regimens as has been recommended recently by the Global Initiative for Asthma and have advised parents to avoid situations where the transmission of the virus to their child and/or themselves can occur (7). This means that these children are currently deprived not only of clinical assessments but also from daily life activities, impacting their physical condition, and social interaction. Healthcare professionals usually keep tight connections with these high-risk asthmatic children during scheduled visits, thereby closely monitoring asthma control and reinforcing skills to prevent a relapse. Studies have clearly shown that these strategies aimed at the early detection of loss of control of asthma are effective (6, 8), especially when parents lack the skills to manage their child's asthma. The COVID-19 crisis however has pressured this care strategy.

## Novel Approaches to Childhood Asthma Care Supported by eHealth

Personalized eHealth strategies using Information and Communication Technology (ICT) and home-monitoring devices could bridge the current lack of care. The COVID-19 crisis offers chances by introducing novel diagnostic and monitoring methods to obtain health status information and maintain close connections with patients at a distance. Monitoring devices assessing heart/breathing rate and oxygen saturation, previously limited to hospital care, have been developed and validated in recent years as wearables suitable for use at home (9). Objective lung function monitoring can be safely performed at home with handheld spirometers and follow trends of airway physiology simultaneously to actual symptoms, revealing skills of perception, and self-management. Smart devices assessing adherence, dose preparation, and even inhalation technique have been developed, providing information on actual compliance (10).

These home-monitoring devices can provide real-time and serial assessments over time, showing that trends of asthma control and risks of severe deterioration can be unobtrusively acquired by combining objective asthma parameters at home (11). This enables healthcare professionals to get a better grip on the episodic course of asthma and therefore makes remote monitoring appropriate for pediatric asthma care.

Despite these technological advances, few eHealth studies have been conducted in childhood asthma care (12), and the widespread application of eHealth is far from our daily clinical practice. However, eHealth studies show promising results (13) and could disrupt care for several reasons: home monitoring measurements, which are acquired when symptoms actually occur at home, can provide potentially more information compared to time-based scheduled visits when healthcare professionals have to rely on patient/parent report and recall. Another advantage of monitoring asthma parameters at home is that it facilitates early medical interventions with inhalers at home rather than by a nebulizer in the hospital. This is important as aerosolization enhances the spread of the COVID-19 virus and the possible transmission to healthcare workers (7).

## Observations and Experiences With Home Monitoring Devices

Before the outbreak of COVID-19, we already introduced the use of home monitoring devices as part of our daily clinical care for children with asthma with a high medical consumption (frequent hospitalizations, emergency visits, *etc*.) to prevent a relapse. During a 6-month exploratory eHealth program, we stopped the scheduled visits and started to closely monitor patients with a web-based eHealth application (Puffer-app) to improve the control of asthma. Through the app, patients could present and discuss symptoms, share symptom videos, ask questions, and share lung function results with their healthcare providers when needed. Moreover, for global monitoring, it was advised to provide a weekly update. Healthcare professionals provided a set of child-friendly monitoring devices for home assessment, containing a handheld spirometer, an oxygen saturation sensor for children who are not able to adequately perform spirometry, and smart inhaler sensors to digitally monitor compliance to medication regimens and inhalation techniques. Monitoring data were sent to a portal and were screened twice daily. Protocols for responses to presented symptoms were followed, which were based on asthma guidelines. If necessary, swift interventions could be initiated by parents and/or patients from a distance, without needing a visit to the hospital.

The data on the Puffer-app application was stored with encryption to protect the privacy of the sensitive data of the patient. Moreover, patients could edit or remove the data at any moment as governed by the Dutch Data Protection Act. The interpretation of the eHealth data, the communications, and the actions that were taken were reported in the electronic health record to guarantee a clean continuation of care after the eHealth program or in case of emergency care. To further protect their safety, all participants were explicitly instructed to not wait for online communication in case of emergency asthma exacerbations and to pursue the regular paths within the healthcare system. Both the medical manager of the pediatric department as well as the board of the hospital approved the use of remote assessment. Children and parents provided informed consent to voluntarily participate and to use the data for the purposes of an exploratory program.

The parents had a high willingness to participate in the technology-supported care program, and the vast majority finished the 6-month study, showing that this innovative asthma care supported by home measurements is feasible and embraced by patients. The eHealth care was beneficial to the majority of asthmatic children in terms of healthcare outcomes and healthcare utilization as it led to a reduction in healthcare utilization, an increase in the self-management level, and improved medication adherence.

In our experience, the insight and the transparency in children's health created by this novel method resulted in the following advantages: (1) suspected causes for loss of control of asthma, such as non-compliance, which is difficult to identify with certainty at “well” visits, became easily manifested with the support of technology, (2) cues of loss of control of asthma could be identified by close home monitoring, (3) communication had a high quality as it was “informed communication” based on the shared observation of actual symptoms in relation to actual compliance and comorbidity and not on recall of symptoms, (4) education, instruction, and reinforcements had a high impact on children and parents as they were provided during the symptoms and not at scheduled visits when information had less direct relevance, (5) patients felt empowered as they were supported by monitoring devices and had the opportunity to seek professional advice at any time with low accessibility, fueling their confidence, and (6) healthcare professionals felt safe and comfortable to provide care at a distance as they could rely on objective home monitoring parameters that they otherwise could have not made available. Moreover, they were able to assess the child serially over time, including the efficacy of the therapeutic interventions.

When the COVID-19 crisis started and scheduled visits were restricted, we already finished the program. To maintain close connections at a distance for these high-risk children during the COVID outbreak, we restarted the program to provide quality care despite the situation.

## Case Report

An 8-year-old asthmatic boy, a poor perceiver, who was monitored in our restarted eHealth program, sent us a message of having a runny nose while his father was sick with a probable COVID-19 infection according to the GP. We advised to isolate the father from the boy and provide a lung function, which was slightly lower than his normal FEV_1_ [85 percent predicted (% pred) compared to his regular FEV_1_ measurements between 85 and 95% pred]. Intranasal saline as needed was added to his nasal steroids. On the day after, he felt slightly dyspneic and blew a FEV_1_ of 74% pred. We reviewed the therapy adherence, which was adequate (>90%) and advised to use a rescue reliever as needed and to follow up with lung function measurements.

**Figure 1:**
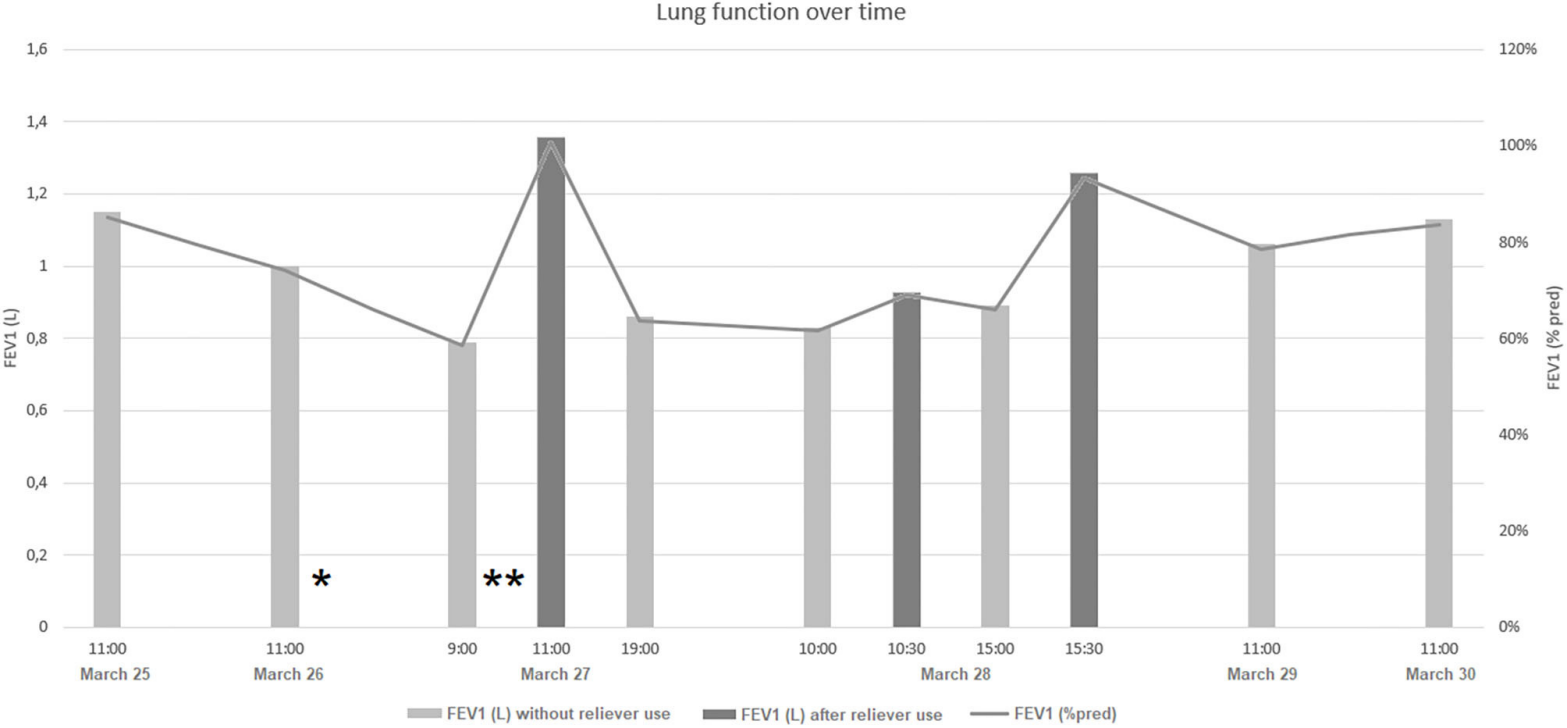
Case report data showing the lung function measurement over time. *The moment that reliever medication as needed was started. **The moment that personal action plan (6 × 400 μg salbutamol daily) was started.

On the next day (March 27), he felt dyspneic, had a temperature of 37.5°C, and coughed a lot; his lung function had worsened to FEV_1_ 58% pred (Figure 1). We asked for a symptom video, which showed a child with an asthma attack: hyperinflation, thoracic retractions, a respiratory rate of ± 30 breaths per min, irregular respiration, and a productive cough. We advised to start with his personal action plan (six times daily of 400 μg salbutamol) and asked to provide a lung function after reliever use. The lung function response to reliever use was significant to FEV_1_ 99% pred, although short-lived. In consultation with the parents, we decided to continue monitoring at home as there was no further decline in the before-reliever lung function measurements during the day and he was responding well to the reliever medication.

On the next morning, we got a message that the boy slept better than on the previous nights. The before-reliever lung function measurement was comparable with the day before. The after-reliever lung function showed an improvement of 12%; however, the flow volume curve was erratic, indicating the clearance of mucus. Over the day, his mother let us know that her son was recovering. He became a bit more active and was less dyspneic. On the days after, the action plan was tapered off and lung function was monitored, which showed a gradual recovery. The working diagnosis was asthma exacerbation due to a viral respiratory infection, possibly COVID-19.

This case report shows that technologically supported eHealth can facilitate safe acute care at a distance and prevent the use of nebulizers and oral steroids. Even though the parents were worried due to the suspected COVID-19 infection of the father and the progressive symptoms of their poorly perceiving son, they relied on lung function monitoring and close supervision. The healthcare providers also felt safe as they were reassured that pre-reliever lung function was stabilized, and there was a post-reliever response.

## Discussion

### Implementation Considerations: What COVID-19 Taught Us

eHealth is a catch-all concept of care using home monitoring devices, communication tools, and ambulatory ICT to organize care. Despite recent validations of eHealth strategies and devices for use at home (14) and the promising results of a few eHealth studies conducted in childhood asthma (13, 15), the actual uptake of eHealth programs into daily clinical practice is scarce. Published eHealth studies are difficult to compare due to heterogeneity in study endpoints and designs which are usually specifically tailored to healthcare systems and cultural backgrounds.

A recent study suggested that a key factor for the successful implementation of eHealth is the alignment of the needs, values, goals, and urgency of both the patients and the healthcare professionals within the specific healthcare system (16). The COVID-19 crisis testified on the importance of these factors as conversion to distant care could be rapidly implemented once societal and individual goals were aligned and a sense of urgency had evolved. COVID-19 seems to have induced a high intrinsic motivation in many patients to convert to distant care and rely on technology at home, whereas before COVID-19, patients who may have been extrinsically motivated by healthcare providers to comply with eHealth for a limited period of time in confined eHealth projects were selected.

COVID-19 was also an eyeopener for healthcare professionals with a more conservative attitude toward eHealth as it revealed the strengths of distant care. Another important lesson learned from the COVID-19 crisis, which was also previously noticed in literature (11, 17), was that we can learn from and tease out relevant data that are acquired worldwide. This principle can help develop eHealth programs adapted to specific healthcare environments and prevent the loss of important information.

More general challenges, such as unobtrusiveness of monitoring, efficacy of tools, and safety and privacy of data, naturally also need to be addressed (13). eHealth platforms should enable users to make individual well-considered choices on levels of privacy, and data should be sanitized and anonymized before encrypted storage to ensure the safe use of personal medical data. Furthermore, the eHealth package should be lean, flexible, and proportional to the severity of the disease and focused at previously identified individual pitfalls to maximize usability and engagement and minimize burden to the child. Gamification approaches may help to take the user engagement in children to a higher level.

eHealth care is characterized by its preventive aspect, aiming to reduce medical consumption by timely interventions. Naturally, for the scalability of eHealth programs, there needs to be a balance between the costs of implementation and the costs due to medical consumption. In recent years, this balance has been gradually shifting toward care at a distance as validated devices have become more affordable, while the costs of hospital care have been rising. However, structural reimbursement is needed to cover additional eHealth expenses and efforts. Otherwise, combined with the loss of revenue from decreasing medical consumption, the financial motive will hamper healthcare institutes to innovate.

### New Approaches to Research and Implementation

The technologically supported endpoints of the study could improve research quality as it can measure the efficacy and the efficiency of therapy more objectively in real life and can address recognized pitfalls of asthma care, such as therapy compliance and perception of asthma symptoms. Previous studies may have over- or underestimated the efficacy of therapies as there were no tools to track these important issues.

Technology also facilitates personalized prognostic medicine by multi-modal monitoring using clinical and real-world data with patient-specific information from health records or environmental data and clustering or prediction modeling. In this way, we may predict the group-based and the individual decline of asthma control and allow teasing out the relative contributions of each intervention tool in the efficacy of eHealth asthma care systems (11, 17). These individual tools could then be offered as part of a modular system to different healthcare environments, preventing generalizations about the efficacy of eHealth programs, and accelerate the validation and the implementation of eHealth asthma care programs for targeted healthcare systems.

We strongly believe that future steps to accelerate eHealth implementation in pediatric asthma care include the further development of multi-modal eHealth platform technologies adapted to the specific healthcare system. We should build upon knowledge from and contributing to worldwide shared data repositories and common data standards to enable personalized prognostic medicine that would facilitate evidence-based eHealth-supported clinical guidelines.

## Conclusion

The COVID-19 era accelerates us to novel methods to stay in close connection with our high-risk asthma children to prevent their deterioration. Technology-supported care at home can complement scheduled hospital evaluations and should be tailored to the individual child and healthcare system.

## Data Availability Statement

The original contributions presented in the study are included in the article/Supplementary Material, further inquiries can be directed to the corresponding author/s.

## Ethics Statement

Written informed consent was obtained from the minor(s)' legal guardian/next of kin for the publication of any potentially identifiable images or data included in this article.

## Author Contributions

MK and BT contributed to the conceptualization and design of the article. MK, MT, and BT drafted the initial manuscript. MT, PL, and SR reviewed and edited the manuscript. All authors contributed to the article and approved the submitted version.

## Conflict of Interest

The authors declare that the research was conducted in the absence of any commercial or financial relationships that could be construed as a potential conflict of interest.
